# An inflammatory paradox: strategies inflammophilic oral pathobionts employ to exploit innate immunity via neutrophil manipulation

**DOI:** 10.3389/froh.2024.1413842

**Published:** 2024-06-11

**Authors:** Dustin L. Higashi, Hua Qin, Christina Borland, Jens Kreth, Justin Merritt

**Affiliations:** ^1^ Division of Biomaterial and Biomedical Sciences, Oregon Health and Science University, Portland, OR, United States; ^2^ Department of Molecular Microbiology and Immunology, Oregon Health and Science University, Portland, OR, United States

**Keywords:** inflammation, pathobionts, neutrophils (PMNs), pathogenesis, microbiome & dysbiosis, innate immunity

## Abstract

Inflammatory dysbiotic diseases present an intriguing biological paradox. Like most other infectious disease processes, the alarm bells of the host are potently activated by tissue-destructive pathobionts, triggering a cascade of physiological responses that ultimately mobilize immune cells like neutrophils to sites of active infection. Typically, these inflammatory host responses are critical to inhibit and/or eradicate infecting microbes. However, for many inflammatory dysbiotic diseases, inflammophilic pathobiont-enriched communities not only survive the inflammatory response, but they actually obtain a growth *advantage* when challenged with an inflammatory environment. This is especially true for those organisms that have evolved various strategies to resist and/or manipulate components of innate immunity. In contrast, members of the commensal microbiome typically experience a competitive growth disadvantage under inflammatory selective pressure, hindering their critical ability to restrict pathobiont proliferation. Here, we examine examples of bacteria-neutrophil interactions from both conventional pathogens and inflammophiles. We discuss some of the strategies utilized by them to illustrate how inflammophilic microbes can play a central role in the positive feedback cycle that exemplifies dysbiotic chronic inflammatory diseases.

## Introduction

Inflammation is a natural and necessary biological response to infection, injury, and/or antigen exposure. Clinically, the outward symptoms of inflammation include localized heat, redness, edema, pain, and the loss of proper function to the affected area. On a cellular level, the inflammatory process is highly complex and nuanced, but its overarching goal is to eliminate the insulting agent, remove damaged cells, and initiate wound healing.

**Figure 1:**
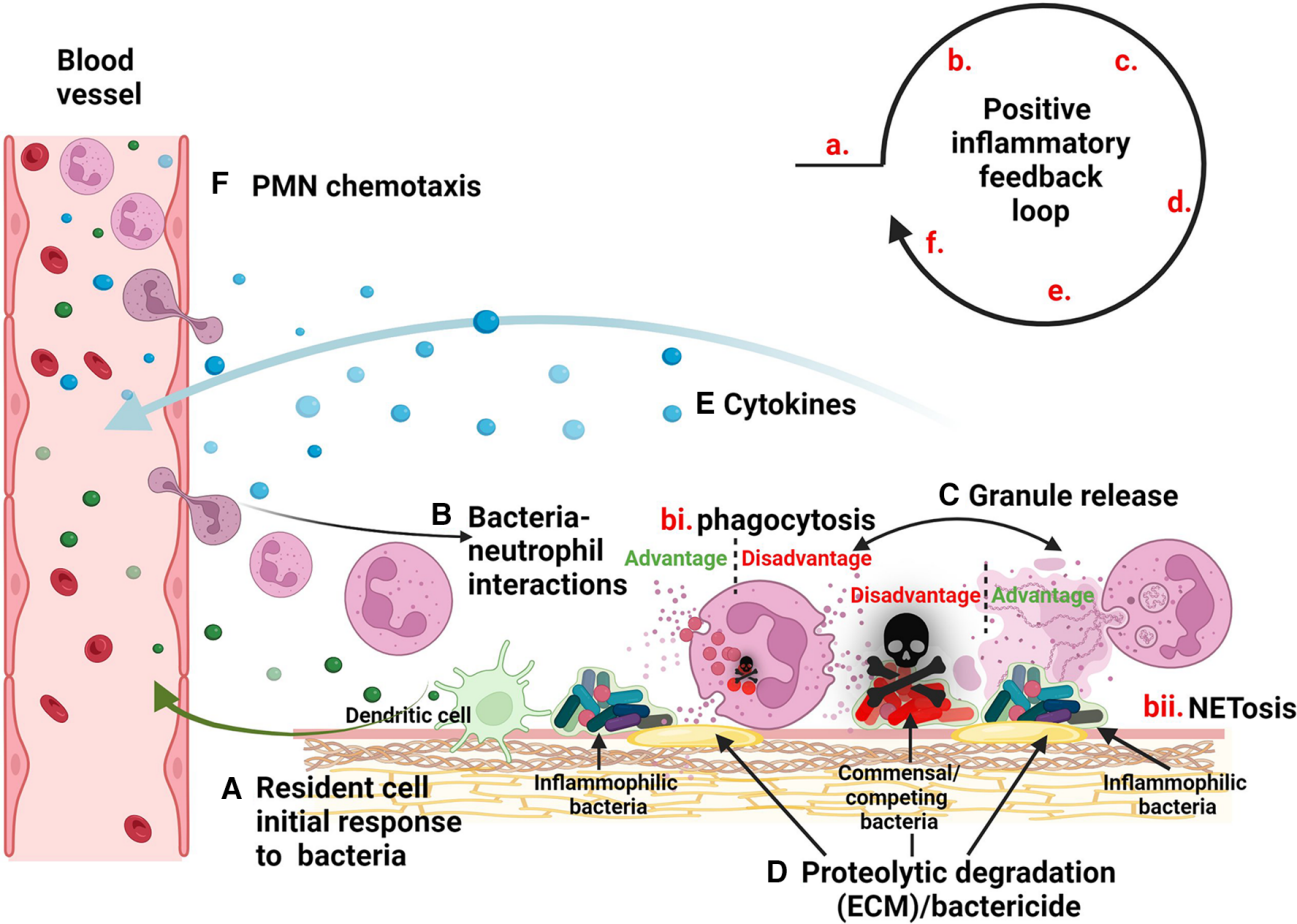
Model of inflammatory dysbiotic disease. (**A**) Inflammophilic bacteria evoke the migration of neutrophils to the site of infection via signaling through resident cells such as dendritic cells. (**B**) Neutrophils mobilize to sites of infection where they encounter bacteria leading to processes such as (bi) phagocytosis and degranulation or (bii) NETosis. (**C**) Granule release causes the (**D**) breakdown of the ECM as well as the elimination of competing micobes. (**E**) Bacteria stimulate neutrophils to release cytokines, (**F**) providing a positive feedback loop for mobilization of more neutrophils.

Inflammation is a crucial protective response of the body when functioning properly. However, its dysregulation can result in an array of diseases, such as atherosclerosis, diabetes, irritable bowel syndrome, cancer, periodontal disease, and others (1, 2). Interestingly, in many of these cases, local inflammation is associated with a dysbiotic shift in the microbial community of the affected site (3). These population shifts not only serve to exacerbate pathogenesis, but they may also serve as an essential step in the sequence of events leading to the downward spiral of chronic inflammation (Figure 1).

During health, the oral microbiome is typically enriched in commensal species, often organisms from genera such as *Neisseria*, *Actinomyces*, *Rothia*, *Corynebacterium*, and *Streptococcus*. These populations promote eubiosis with the host by limiting acidification of dental plaque, antagonizing the growth of pathobionts, and protecting the gingiva by dampening inflammation (4). For periodontitis, which is a chronic inflammatory disease that damages the supporting structures surrounding the tooth, disease is characterized by a dysbiotic shift in favor of inflammophilic microbes (i.e., those that thrive within an inflammatory environment). Genera such as *Parvimonas*, *Porphyromonas*, *Fusobacterium*, *Prevotella*, *Tannerella, Treponema*, and others predominate concurrently with chronic gingival inflammation, resorption of the alveolar bone, and the destruction of the connective tissues supporting the teeth (5–8). Presently, we only have a tenuous understanding of the polymicrobial aspects of dysbiotic immunopathology, but studies hint at highly coordinated and evolved processes.

For oral dysbiotic communities, members exhibit an exquisite reliance upon polymicrobial synergism to promote and maintain chronic inflammation. Through cooperation, microbes within these communities are able to enhance colonization, optimize nutrient acquisition, provide protection from environmental insults, and bolster pathogenicity (9). Accordingly, a number of studies have demonstrated how such synergism may occur among prominent inflammophilic oral pathobionts. For instance, *Parvimonas micra* has been shown to coaggregate with *Treponema denticola*, *Porphyromonas gingivalis*, and *Fusobacterium nucleatum* (10–12). Furthermore, *P. micra* can utilize soluble factors from these organisms to enhance its own growth, and can in turn, release factors that increase biofilm formation and the growth of *P. gingivalis* and *F. nucleatum* (11). Mixed biofilms of *P. micra* display increased resistance to sodium hypochlorite treatment as compared to single species biofilms (13). Coinfection with *Prevotella intermedia* was shown to increase the virulence of *P. micra* in a murine abscess model (14). *P. micra* may also influence the production of virulence factors from other members of the community. For example, *P. micra* is a potent stimulator of *P. gingivalis* gingipains, which are secreted proteases that serve as key virulence factors involved in host cell adhesion, nutrient acquisition, biofilm development, and immune evasion (15).

The strength of the symbiotic relationships between inflammophilic microbes is demonstrated by their frequent, if not typical, associations across numerous diseases. Indeed, many of the same species enriched in periodontitis are also highly prominent in acute infections such as odontogenic abscesses (16, 17). In malignant tumors, studies have demonstrated an enrichment of many of the same genera as those found in oral dysbiotic diseases, especially *Parvimonas*, *Fusobacterium*, and *Prevotella* (18–21). Co-infections at typically sterile sites throughout the body illustrate how members within these groups can survive extensive journeys together to establish new infections at distant extraoral sites (22, 23).

While it is now evident that inflammophilic bacterial communities are able to survive and thrive in inflammatory environments, the specific roles of each member remain largely unknown in the polymicrobial context. To understand the mechanisms of polymicrobial synergism in inflammatory dysbiotic disease, it is necessary to first reveal how individual species within these communities impact host immunity. Once this baseline understanding is established, the field can then compare how polymicrobial synergism modifies the expressed phenotypes of more complex assemblages. With this in mind, we recently presented new findings regarding neutrophil interactions with the inflammophilic pathobiont *P. micra* while at the 3rd International Conference on Oral Mucosal Immunity and Microbiome. However, these results represent only a small component of a much larger puzzle. Therefore, the following sections will describe some prominent examples of the interplay between neutrophils and inflammophilic microbes as well as conventional pathogens to demonstrate the broader implications for inflammatory dysbiotic disease, especially among complex polymicrobial communities. We discuss how aspects of inflammation provide a selective advantage, particularly for the inflammophilic members within these communities.

### The neutrophil

Polymorphonuclear leukocytes (neutrophils) are a central component of the innate immune system and make up the majority of white blood cells in humans. As one of the first defenders to sites of infection, neutrophils are key mediators that influence the host response and play a central role in pathogenesis and the resolution of disease (24–26). In recent years, it has become increasingly apparent that neutrophils engage in extensive crosstalk with both immune and non-immune cells (27). They have been shown to induce the production and secretion of cytokines from endothelial and epithelial cells (28). Neutrophils induce the activation and migration of macrophages and dendritic cells. They can also facilitate the activation, inhibition, and differentiation of T cells as well as promote B cell expansion (29). Amazingly, multiple studies have reported that T and B cell activation can be *directly* facilitated by a specialized subset of neutrophils capable of presenting antigens via both MHC class I and class II (30, 31).

### Granules

Neutrophils are mini-armories, possessing a vast array of defensive and offensive weaponry to battle microbial infections. These include multiple types of granules—tiny membrane-bound organelles within neutrophils containing numerous components like complement receptor, lactoferrin, metalloproteases, lysozyme, elastase, collagenase, myeloperoxidase, defensins, and more (32). These cargos are intimately connected with almost every aspect of neutrophil biology and have a myriad of functions, including the digestion of microbes, nutrient sequestration, antimicrobial activity, and modulation of the adaptive immune response.

### Extracellular degranulation

Granule contents are not only toxic to microbes, but they can also damage host tissues following extracellular degranulation (i.e., release of neutrophil granule contents to the extracellular milieu). Certain infecting microbes are particularly adept at provoking neutrophil degranulation. For example, a methicillin-resistant strain of *Staphylococcus aureus* was shown to utilize a phenol-soluble modulin to induce the phosphoinositide 3-kinase (PI3 K) dependent degranulation of neutrophils (33). *Streptococcus pyogenes* induces degranulation through an M protein-fibrinogen complex (34). *Filifactor alocis* engagement of TLR2 induces the release of secondary specific granules (35). In contrast, some microbes actively suppress neutrophil degranulation. *Chlamydia trachomatis* produces a protease-like activating factor (CPAF) that cleaves formyl peptide receptor 2 (FPR2) on neutrophils, preventing PI3K-induced degranulation. Importantly, a *C. trachomatis* CPAF mutant was demonstrated to be highly susceptible to neutrophil granules, unlike its parental wild-type (36).

How does extracellular degranulation influence disease? It is possible that certain microbes may actively induce granule release as an adjunctive strategy to impair susceptible competitors, usurping the antimicrobial arsenal of the host. For example, endodontic infections are seeded by the oral biofilm, yet persistent pressure from a neutrophil-rich immune response results in an abscess community composition that exhibits little resemblance to its original inoculum (16, 37). In particular, much of the commensal microbiome antagonists of oral pathobionts become depleted within the abscess environment, whereas these same species comprise a prominent fraction of normal dental plaque communities (38). Inducing degranulation could also provide nutrition for bacteria. Granules contain proteases that target not only bacteria but also extracellular matrix (ECM) proteins such as elastin and collagen. Release of these enzymes into the ECM facilitates the breakdown of host proteins into smaller peptides, making them available for bacterial consumption. It is worth noting that many of the commonly encountered inflammophilic pathobionts are also slow-growing fastidious organisms that prefer to metabolize amino acids, in contrast to the typical commensal species, which are primarily fast-growing saccharolytic organisms. Thus, inflammatory tissue destruction naturally provides a continual source of soluble free peptides, which are the preferred carbon sources for key inflammophilic organisms (39, 40). In the context of host signaling, degranulation is also pro-inflammatory, which serves to further promote an inflammatory growth environment favorable to inflammophilic microbes and unfavorable to many commensal species.

### Phagocytosis and granule-phagosome fusion

Neutrophils are professional phagocytes that possess an uncanny capacity to engulf and eliminate bacteria. After ingestion, neutrophil granules fuse with the bacterial phagosome resulting in the release of toxic bactericidal compounds. While an effective means to eliminate unwanted invaders, some pathogens have evolved mechanisms to circumvent these defenses.

One strategy involves targeting neutrophil phagocytosis at its initial stages. *P. gingivalis* signals neutrophils through TLR2 and C5aR leading to the proteasomal degradation of MyD88 and activating an alternate TLR2-Mal-PI3 K pathway that inhibits phagocytosis (41). *Neisseria meningitidis* avoids complement-mediated phagocytosis by binding host Factor H (42), whereas *Yersinia pestis* produces a F1 capsule which directly inhibits phagocytosis (43).

Following phagocytosis, the bacteria-containing phagosome matures through fusion with antimicrobial granules, resulting in the killing of the ingested microbe. A number of bacteria are able to prevent killing after ingestion by neutrophils. *Neisseria gonorrhoeae* can alter phase-specific production of the Opa protein to prevent the accumulation of primary granule proteins, thus enhancing their survival (42). The *S. pyogenes* M protein has been shown to selectively prevent the fusion of primary granules with phagosomes (44). Live *F. alocis* prevents primary granule recruitment to its phagosome compared to heat killed bacteria (45) and *Mycobacterium smegmatis* phagosomes fail to fuse with primary granules (46).

Microbes may benefit from the manipulation of phagocytosis and granule fusion in multiple ways. First, they avoid killing by preventing the engagement of neutrophil granules altogether. Extracellular bacteria remain unharmed while ingested bacteria are afforded protection from external threats. Furthermore, these intracellular bacteria could conceivably utilize the migrating neutrophil to disseminate to new sites of infection.

### Neutrophil extracellular traps (NETs)

Neutrophils can also produce extracellular traps (NETs) in response to infection. Through a process called NETosis, neutrophils elaborate structures comprised of extracellular fibers of chromatin and granular proteins that are able to trap and kill pathogens (47). While an effective defense against most microbes, a number of bacteria have evolved strategies to subvert NET production or function (48).

*S. pyogenes* produces the protease SpyCEP which cleaves IL-8 to inhibit NET formation (49). *S. aureus* signaling through the phosphatase Wip1, inhibits calcium signaling to suppress NETosis (50). *Pseudomonas aeruginosa* adsorbs host sialoglycoproteins to engage siglec-9 and inhibit NET formation. *S. pyogenes* also engages siglec-9 for a similar outcome, but does so via its production of a high molecular weight hyaluronan capsule (51, 52). In group B *Streptococcus*, the cell wall β-protein impairs NET formation through engagement of siglec-5 (53).

In the presence of NETs, bacteria have evolved other means of survival. *Prevotella intermedia* can degrade NETs through the activity of NucA and NucD nucleases (54). Likewise, *S. pyogenes* produces Sda1 and SpnA nucleases to digest NETs and promote survival (55, 56). *P. gingivalis* produces the enzyme peptidylarginine deiminase (PPAD), which was shown to citrullinate histone H3 and the antimicrobial peptide LP9, facilitating bacterial escape and survival (57). Furthermore*, P. gingivalis* gingipain induces the activation of protease-activated receptor-2 (PAR-2) to induce NETs that lack bactericidal activity and also stimulate *P. gingivalis* growth (58).

The manipulation of NETosis and NETs by microbes demonstrates a highly evolved arms race which seems to involve most aspects of neutrophil biology. The ability of microbes to use compounds recognized as “self” molecules like hyaluronan and sialoglycoproteins supports signaling through host-specific pathways and demonstrates an ingenious form of molecular mimicry to undermine host defenses. Many microbes naturally promote NETosis as a consequence of their inherent pro-inflammatory nature (48). For those that have evolved ways to resist NET-dependent killing, the formation of NETs themselves may even be beneficial due to its aggravation of the inflammatory response (59). NETs contain many granular components including proteases like elastase. As posited above, the ability of these enzymes to digest the ECM may provide a peptide-rich food source for surrounding microbes, especially organisms that favor amino acid fermentation. Similarly, antimicrobial NETs may also target competing species like commensal organisms, further selecting for inflammophilic community development. NETs may also delay wound healing, which would encourage persistent infections (59).

### Inflammatory signaling

Historically, neutrophils were not considered a major source of signaling molecules and were primarily viewed as strict responders to environment cues such as the chemokines produced by activated epithelial cells (60). However, it is now well established that neutrophils actively secrete numerous signal molecules, such as various cytokines (e.g., TNFα, IL-1β, IL-6, IL-8), colony-stimulating and angiogenic factors, as well as growth factors (61). Neutrophils treated with *Mycobacterium tuberculosis* secrete IL-8 and GRO-α (62). *P. gingivalis*, *Peptoanaerobacter stomatis*, and *F. alocis* induce neutrophil release of TNFα, IL-1β, and IL-1RA (63).

The abundance of neutrophils present in dysbiotic inflammatory infections suggests that bacteria having the ability to regulate anti- or pro-inflammatory signaling cascades have likely tapped into a potent feedback loop to actively modulate inflammation.

## Conclusions and future directions

Inflammatory dysbiosis results from the disruption of normal microbiome ecology, resulting in the overgrowth of normally low abundance pathobionts. The species within these microbial communities synergize to support their survival and growth by actively promoting an ineffective inflammatory response. How do these bacteria thrive in such a hostile environment designed to inhibit bacterial growth? One explanation may lie in the fact that many inflammophilic pathobionts have naturally evolved to reside in such environments. For example, oral pathobionts like *P. micra* typically reside within the gingival sulcus, an environment continually bathed in gingival crevicular fluid (GCF), which is comprised of many innate host defenses like proteolytic enzymes, antibodies, complement, and neutrophils (64). It is conceivable that the same phenotypes allowing pathobionts to persist in the gingival sulcus could prove to be pathogenic to the host when expressed in a different environmental context, especially if such organisms were to achieve high numbers in the community. Studies on microbe-neutrophil interactions provide clues into the types of strategies employed by individual inflammophilic bacteria. It is clear that some microbes are able to commandeer and manipulate neutrophil functions for their benefit. The looming question is whether these same responses observed from individual species still occur similarly in a polymicrobial context. For example, some oral pathobionts provoke neutrophil NETosis, while others inhibit this process. What is the final outcome when these organisms coexist in polymicrobial communities? Is it simply a numbers game, with the greater abundance organism yielding the dominant effect upon surrounding neutrophils or is it an altogether unique neutrophil response that does not resemble the responses to the individual constituents of the community? One could ask a similar question regarding neutrophil production of signal molecules that direct downstream components of the immune response.

These questions portend necessary future investigations of inflammophilic synergism as a crucial next frontier in dysbiotic pathogenesis research. A better understanding of the mechanisms used by inflammophilic microbes to manipulate neutrophils during infection may reveal new therapeutic strategies for the treatment of dysbiotic inflammatory diseases. Indeed, therapeutic approaches targeting neutrophil functions have already been proposed for the treatment of ailments such as cancer, pulmonary disease, and sepsis, with active clinical trials investigating a number of medical indications (65). For inflammatory oral diseases like periodontitis, therapeutic approaches targeting pathobiont-induced neutrophil immunopathologies may ultimately restrict pathobiont growth, thereby reducing inflammation, tissue damage, and bone loss.

## Data Availability

The original contributions presented in the study are included in the article/Supplementary Material, further inquiries can be directed to the corresponding author.
